# Redundancy among risk predictors derived from heart rate variability and dynamics: ALLSTAR big data analysis

**DOI:** 10.1111/anec.12790

**Published:** 2020-08-17

**Authors:** Emi Yuda, Norihiro Ueda, Masaya Kisohara, Junichiro Hayano

**Affiliations:** ^1^ Tohoku University Graduate School of Engineering Sendai Japan; ^2^ Nagoya City University Graduate School of Medical Sciences Nagoya Japan

**Keywords:** ALLSTAR, big data, heart rate variability, mortality, redundancy, relationship mapping

## Abstract

**Background:**

Many indices of heart rate variability (HRV) and heart rate dynamics have been proposed as cardiovascular mortality risk predictors, but the redundancy between their predictive powers is unknown.

**Methods:**

From the Allostatic State Mapping by Ambulatory ECG Repository project database, 24‐hr ECG data showing continuous sinus rhythm were extracted and *SD* of normal‐to‐normal R‐R interval (SDNN), very‐low‐frequency power (VLF), scaling exponent α_1_, deceleration capacity (DC), and non‐Gaussianity λ_25s_ were calculated. The values were dichotomized into high‐risk and low‐risk values using the cutoffs reported in previous studies to predict mortality after acute myocardial infarction. The rate of multiple high‐risk predictors accumulating in the same person was examined and was compared with the rate expected under the assumption that these predictors are independent of each other.

**Results:**

Among 265,291 ECG data from the ALLSTAR database, the rates of subjects with high‐risk SDNN, DC, VLF, α_1_, and λ_25s_ values were 2.95, 2.75, 5.89, 15.75, and 18.82%, respectively. The observed rate of subjects without any high‐risk value was 66.68%, which was 1.10 times the expected rate (60.74%). The ratios of observed rate to the expected rate at which one, two, three, four, and five high‐risk values accumulate in the same person were 0.73 times (24.10 and 32.82%), 1.10 times (6.56 and 5.99%), 4.26 times (1.87 and 0.44%), 47.66 times (0.63 and 0.013%), and 1,140.66 times (0.16 and 0.00014%), respectively.

**Conclusions:**

High‐risk predictors of HRV and heart rate dynamics tend to cluster in the same person, indicating a high degree of redundancy between them.

## 1 INTRODUCTION

Heart rate variability (HRV) and heart rate (HR) dynamics are defined as the quantitative and qualitative features of physiological beat‐to‐beat heartbeat interval fluctuations, respectively (Camm et al., 1996; Dynamic electrocardiography, 2004). The analysis of HRV and HR dynamics has been widely used for autonomic functional assessment (Hayano, 2016; Hayano & Yuda, 2019) and risk stratification (Watanabe, Kiyono, Yamamoto, & Hayano, 2016). Long‐term R‐R interval time series data obtained by 24‐hr ambulatory Holter ECG are the main sources for these analyses, and several indices have been proposed as mortality risk predictors in cardiovascular diseases (Bauer et al., 2006; Bigger et al., 1992; Hayano et al., 2011; Huikuri et al., 2000; Kantelhardt et al., 2007; Kiyono, Hayano, Watanabe, Struzik, & Yamamoto, 2008; Kiyono, Struzik, & Yamamoto, 2007; Kleiger, Miller, Bigger, & Moss, 1987; Peng, Havlin, Stanley, & Goldberger, 1995).

The HRV indices are classified into time‐domain and frequency‐domain indices (Camm et al., 1996). The time‐domain indices consist of various statistical measures of the variations in normal‐to‐normal (N‐N) R‐R interval (R‐R interval of consecutive sinus rhythms). The representatives are the standard deviation of 24‐hr N‐N interval (SDNN) (Kleiger et al., 1987) and deceleration capacity (DC) (Kantelhardt et al., 2007). A decrease in these indices predicts increased mortality risk after acute myocardial infarction (AMI) (Bauer et al., 2006). The frequency‐domain indices are calculated by power spectral analysis of the N‐N interval time series and are quantified as the power of frequency components. Among such components, a reduction in the power of a very‐low‐frequency band (0.0033–0.04 Hz) is the most powerful predictor of post‐AMI mortality (Bigger et al., 1992). The indices of HR dynamics include various nonlinear indices that capture the qualitative feature of fluctuation. Detrended fluctuation analysis (DFA) (Peng et al., 1995) quantifies the scaling exponents of fractal‐like HR dynamics, and a reduction in the short‐term (4–11 beats) exponent (α_1_) is increased mortality risk in post‐AMI patients (Huikuri et al., 2000). The non‐Gaussianity index (λ) quantifies the probability density function for abrupt large HR changes (Kiyono et al., 2007), and an increase in λ predicts increased mortality risk in patients with heart failure (Kiyono et al., 2008) and in those after AMI (Hayano et al., 2011).

Earlier reports advocating new mortality predictors have shown that their predictive power is independent, at least partly, of those reported earlier. However, there are no large‐scale studies that verified systematically the relationships between the predictors, and there are no credible facts as to whether they are independent of each other, or whether there is redundancy among them. In this study, we analyzed their inter‐relationships and examined the degree of redundancy among the major prognostic predictors of HRV and HR dynamics. For these purposes, we used 24‐hr ECG big data obtained from the Allostatic State Mapping by Ambulatory ECG Repository (ALLSTAR) project (Hayano, Kisohara, Ueda, & Yuda, 2020; Hayano et al., 2018).

## 2 METHODS

### 2.1 ALLSTAR database

We obtained 24‐hr ECG data from the ALLSTAR database, which contained 430,169 Holter ECGs recorded between November 2007 and March 2014. The ALLSTAR project has started in 2007 in Japan. The purpose of the project is to build a cumulative database of 24‐hr ambulatory ECG recorded all over Japan.

This study was performed according to the protocol approved by the Ethics Review Committee of Nagoya City University Graduate School of Medical Sciences (No. 709). Additionally, following the Ethical Guidelines for Medical and Health Research Involving Human Subjects (by the Ministry of Education, Culture, Sports, Science and Technology and the Ministry of Health, Labour and Welfare, Japan, December 22, 2014), the purpose and information utilized in this project have been public through the project's homepages (http://www.suzuken.co.jp/product/holter/detail/ and http://www.med.nagoya‐cu.ac.jp/mededu.dir/allstar/), in which opportunities to refuse the uses of information are ensured for the research subjects.

The 24‐hr ECG data in this database were recorded for some clinical purpose(s) by medical facilities and were referred for analysis to three ECG analysis centers (Suzuken Co., Ltd.) located in Tokyo, Nagoya, and Sapporo in Japan. The data were anonymized by the centers and stored with accompanying information, including age, sex, and recording date, time, and location (postal code). Table 1 shows the characteristics of ALLSTAR subjects, including underlying cardiac diseases, cardiovascular risk factors, and medications, obtained from a randomized survey of 73,582 (17%) subjects.

**Table 1:** Characteristics of subjects in ALLSTAR database

	Ratio, %
Cardiac disease	
Coronary artery diseases	4.93
Cardiomyopathy	0.64
Valvular heart diseases	2.36
Congenital heart diseases	0.80
Heart failure	4.03
Arrhythmias	45.67
Cardiovascular risk factors	
Hypertension	37.48
Diabetes	10.29
Dyslipidemia	20.06
Healthy subjects (screening examination)	10.74
Medications	
Calcium antagonists	33.68
Angiotensin II antagonists	26.10
β blockers	8.97
Diuretics	8.46
Nitrates	6.11
Antiarrhythmic drugs	6.03
Antidiabetics	7.69
Hyperlipidemic drugs	20.92
No medication	26.66

Note

Data were obtained from a random sampling survey of 73,582 (17%) subjects. Subjects with multiple diseases or medications are counted repeatedly.

All data were recorded with the Cardy series of Holter ECG recorders (Cardy 2, Cardy 2P, Cardy 203, Cardy 301, Cardy 302 Mini and Max, Cardy 303 pico and Cardy 303 pico+, Suzuken Co., Ltd., Nagoya, Japan), by which multi‐channel ECG data were digitized at 125 Hz with 10 bit (0.02 mV/digit). The digitized data were sent to the analysis centers and analyzed with Holter ECG analyzers (Cardy Analyzer 05, Suzuken Co., Ltd.); the temporal positions of all R waves were determined, the rhythm annotations were given to all QRS complexes, and all errors in the automated analysis were corrected manually by skilled medical technologists. The suspicious outcomes of the analysis have been reviewed by contracted cardiologists.

### 2.2 Data selection

From the ALLSTAR database, 24‐hr ECG data were selected for this study with the following criteria.

Data were included only if all of the following were met:

1. Subject age at ECG recording >20 year
2. The first ECG recording, if there was a repeated recording
3. Record length >21.6 hr (90% of 24 hr), and
4. Cardiac rhythm is in sinus rhythm for >19.2 hr (80% of 24 hr).

Data were excluded if ECG showed at least one of the following:

1. Evidence of artificial pacemaker implantation or
2. Non‐sinus rhythm beats >20% of total recorded beats.

### 2.3 Computations of predictors

We studied the time‐domain and frequency‐domain indices of HRV and nonlinear indices of HR dynamics that are known as the major predictors of increased cardiovascular mortality risk. They were computed by the methods according to the recommended standard (Camm et al., 1996) and to the earlier studies (Iyengar, Peng, Morin, Goldberger, & Lipsitz, 1996; Kantelhardt et al., 2007; Kiyono et al., 2007; Peng et al., 1995). Briefly, from the ECG data, the time series of N‐N intervals, {*NN_i_*} = {*tN_i_* − *tN_i_*_−1_}, where *tN_i_* represents the time of occurrence of the *i‐*th normal sinus beat were derived. For the time domain HRV indices, SDNN was computed as 24‐hr standard deviation of *NN_i_* and DC was computed by the phase rectified signal averaging of the 24‐hr N‐N interval time series (Kantelhardt et al., 2007). For a frequency domain index, we computed VLF power. For this purpose, {*NN_i_*} time series were interpolated by a horizontal step function, resampled at 2 Hz, filtered with a Hanning window, and converted into the frequency domain by a Fast Fourier transform (FFT). The power spectral density was integrated for the power within the VLF band (0.0033–0.04 Hz). The VLF power was transformed into a natural logarithmic value to normalize the distribution. For the nonlinear indices, we calculated the fractal correlation properties of HR dynamics using the DFA method and measured the short‐term (4 to 11 beats) scaling exponents(α_1_) (Iyengar et al., 1996; Peng et al., 1995). We also calculated the non‐Gaussianity index of λ setting the scale at 25 s (λ_25s_) according to the previous study (Hayano et al., 2011).

### 2.4 Assessment of inter‐relationships between predictors

To assess the relationships between the predictors, Pearson's correlation coefficients (*r*) were calculated and the similarities between them were defined as (1 − |r|). To visualize the inter‐relationships, a relationship map of the predictors, where the distances between them best match with the similarities between them, was obtained by exhaustive computer searching through all possibilities. The mutual independency of predictors was also assessed by the squared multiple correlation coefficient (*R* ^2^) calculated by multiple regression analysis of predictor with all other predictors as explanatory variables.

### 2.5 Assessment of redundancy

To assess the redundancy between their predictive powers, the values of each predictor were dichotomized into high risk and low risk using the cutoff reported in earlier studies to predict post‐AMI mortality. We used SDNN < 65 ms (Huikuri et al., 2000), DC ≤ 2.5 ms (Bauer et al., 2006), VLF < 5.75 ln(ms^2^) (Huikuri et al., 2000), DFA α_1_ < 0.75 (Huikuri et al., 2000), and λ_25s_ > 0.6 (Hayano et al., 2011) as high risk. Note that SDNN, DC, VLF, and DFA α_1_ represent high risk when the values are below the thresholds, while λ_25s_ represents high risk when the value is above the threshold (Hayano et al., 2011). Then, the appearance rate of the high‐risk value for each predictor was measured. Using the obtained rate, the expected probability that 0 to 5 high‐risk predictors out of 5 would appear in the same person was calculated under the assumption that the predictors are independent of each other. Then, we compared the expected and observed risk clustering rates.

### 2.6 Statistical analysis

SAS program package (SAS Institute) was used for statistical analyses. The Freq, Corr, and Reg procedures were used for assessing occurrence rates, correlation coefficients, and multiple correlations, respectively. *p* < .05 was used for the criteria of statistical significance.

## 3 RESULTS

### 3.1 Characteristics of the sample population

From 405,911 ECG data in the ALLSTAR database of subjects aged ≥ 20 years, 84,591 (20.84%) were excluded due to repeated recordings. Of the remaining 321,220 data, 21,014 (6.54%) due to insufficient data length, 20,034 (6.23%) due to atrial fibrillation or flatter, 13,658 (4.25%) due to frequent > 20% ectopic beats, and 1,323 (0.41%) due to implanted pacemaker were excluded, and 265,291 data that met all criteria were finally used in this study. Subjects of the used data were aged 65 ± 16 (mean ± *SD*) years, 116,554 males, and 148,737 females.

### 3.2 Inter‐relationships between predictors

Table 2 showed the means and *SD* of the predictors from HRV and HR dynamics in the 24‐hr ECG data, and Table 3 presents correlation coefficients among them. A close positive correlation was observed between SDNN and VLF and mild to moderate positive correlations among SDNN, DC, VLF, and DFA α_1_. In contrast, λ_25s_ showed no significant correlation with SDNN and moderate negative correlations with DC, VLF, and DFA α_1_.

**Table 2:** Distribution of risk predictors in the ALLSTAR big data (*n* = 265,291)

Predictor	Mean	Median (IQR)
SDNN, ms	137 ± 43	134 (108–162)
DC, ms	6.03 ± 2.30	6.02 (4.72–7.37)
VLF, ln(ms^2^)	7.07 ± 0.85	7.10 (6.59–7.60)
DFA α_1_	1.05 ± 0.27	1.08 (0.87–1.25)
λ_25s_	0.55 ± 0.11	0.53 (0.47–0.60)

Abbreviations

DC, deceleration capacity; DFA, detrended fluctuation analysis; SDNN, *SD* of normal‐to‐normal R‐R (NN) intervals during 24 hr; VLF, very‐low‐frequency (0.0033–0.04 Hz) power.

**Table 3:** Correlation coefficients among risk predictors (*n* = 265,291)

	SDNN	DC	VLF	DFA α_1_	λ _25s_
SDNN	‐	0.30	0.71	−0.00027*	−0.019
DC	0.30	‐	0.46	0.39	−0.30
VLF	0.71	0.46	‐	0.18	−0.20
DFA α_1_	−0.00027*	0.39	0.18	‐	−0.20
λ_25s_	−0.019	−0.30	−0.20	−0.20	‐

Note

All correlation coefficients are statistically significant except those marked with *. Abbreviations are explained in the footnote to Table 2.

Table 4 shows the independence of each predictor from the others. A significant regression model was obtained for all predictors. The explained variances by the other predictors (*R* ^2^), whose small values represent independence from the others, were the smallest for λ_25s_ and the largest for VLF.

**Table 4:** Mutual multiple regression analysis with other predictors (*n* = 265,291)

Predictor	Multiple regression analysis by the other predictors		
	*F* value	*p*	*R ^2^*
SDNN	53,675	<.0001	.524
DC	26,084	<.0001	.349
VLF	69,020	<.0001	.586
DFA α_1_	11,060	<.0001	.185
λ_25s_	7,032	<.0001	.126

Note

Abbreviations are explained in the footnote to Table 2.

**Figure 1:**
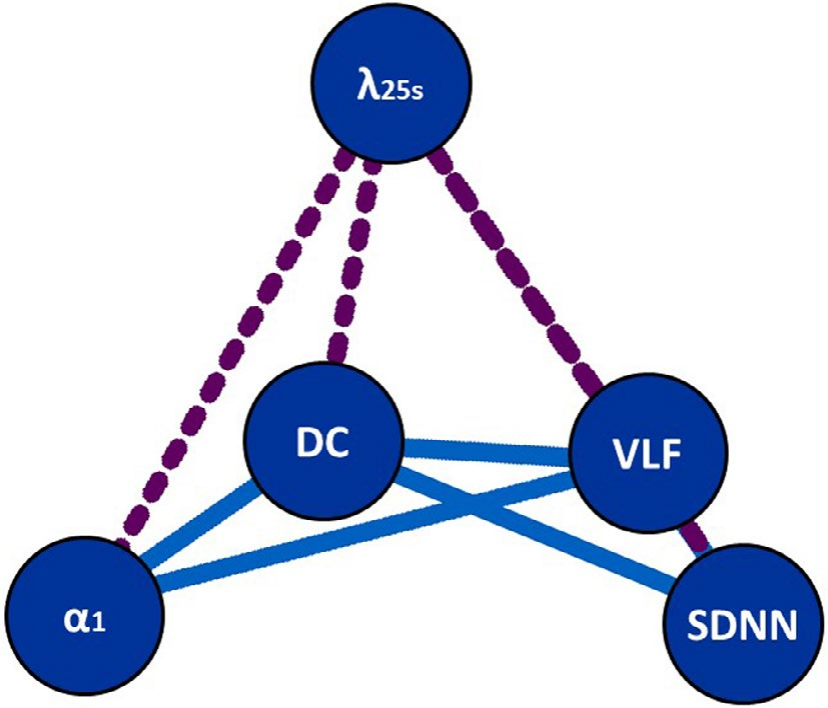
Relationship mapping of HRV and HR dynamics indexes where the distance between the indexes best match with the similarity between the indices (1 – |r|). The placement of indices was determined by a computer search through all possibilities. Dotted lines indicate a negative correlation. The abbreviations are explained in the footnote to Table 2

Figure 1 is the relationship mapping of predictors where the distance between the predictors best match with the similarity between them. Although this figure represents only the relative similarity between indices, predictors that reflect the quality and quantity of HR fluctuations are separated in the upper left and lower right, and those that reflect sympathetic and parasympathetic functions are separated vertically.

### 3.3 Redundancy of predictive powers

Table 5 shows the observed rate of the low‐risk and high‐risk subjects for each predictor. From these rates, the expected rates of multiple high‐risk predictor items accumulating in the same person were calculated under the assumption that the predictors are independent of each other. As shown in Table 6, the observed rate of subjects with no high‐risk item was 66.68%, which was 1.10 times the expected rate (60.74%). The observed rate was lower (0.73 times) than the expected rate for subjects who had only one high‐risk item. For the subjects with multiple (two to five) high‐risk items, the observed rates were always greater than the expected rate, and for the subjects with all of the five high‐risk items, the observed rate was 1,140.66 times the expected rate.

**Table 5:** Observed rate of subjects with low‐risk and high‐risk values (*n* = 265,291)

Predictor with cutoff value	Observed rate (%)	
	Low‐risk case	High‐risk case
SDNN < 65 ms	97.05	2.95
DC ≤ 2.5 ms	97.25	2.75
VLF < 5.75 ln(ms^2^)	94.11	5.89
DFA α_1_ < 0.75	84.25	15.75
λ_25s_ > 0.6	81.18	18.82

Note

Abbreviations are explained in the footnote to Table 2.

**Table 6:** Expected and observed rates of subjects by the number of high‐risk values

Number of high‐risk values	Expected rate (%)	Observed		Ratio*
		Case	Rate (%)	
0	60.74	176,894	66.68	1.10
1	32.82	63,922	24.10	0.73
2	5.99	17,412	6.56	1.10
3	0.44	4,951	1.87	4.26
4	0.013	1,683	0.63	47.66
5	0.00014	429	0.16	1,140.66

* Ratio of observed rate to expected rate.

Table 7 shows the expected and observed rates of all possible combinations of high‐risk predictor items. For all combinations of four high‐risk items, the observed rates were > 19 times higher than the expected rates. On the other hand, the observed rate of subjects with a single isolated high‐risk value of SDNN, DC, α_1_, or λ_25s_ was lower than the expected rate.

**Table 7:** Expected and observed rates of subjects by the combination of high‐risk values

Combination of high‐risk values	Expected rate (%)	Observed		Ratio*
		Case	Rate (%)	
SDNN + DC+VLF + α_1_	0.00061	538	0.20	331.61
SDNN + DC+VLF + λ_25s_	0.0023	435	0.16	72.44
VLF + DC+α_1_ + λ_25s_	0.0047	400	0.15	32.31
SDNN + DC + VLF	0.0098	759	0.29	29.30
SDNN + VLF + α_1_ + λ_25s_	0.00076	46	0.017	22.87
SDNN + DC+α_1_ + λ_25s_	0.0050	264	0.10	19.86
VLF + α1 + λ_25s_	0.025	883	0.33	13.34
VLF + α_1_	0.022	533	0.20	9.30
VLF + λ_25s_	0.11	2,631	0.99	9.21
SDNN + DC + α_1_	0.0033	68	0.026	7.84
DC + VLF + λ_25s_	0.40	8,240	3.11	7.79
SDNN + VLF + α_1_	0.075	1,457	0.55	7.37
DC + VLF + α_1_	0.020	366	0.14	6.85
VLF	1.72	23,442	8.84	5.14
SDNN + VLF + λ_25s_	0.012	90	0.034	2.80
SDNN + DC + λ_25s_	0.080	534	0.20	2.52
DC + VLF	0.32	1754	0.66	2.06
SDNN + DC	0.35	1,780	0.67	1.94
SDNN + VLF	0.052	255	0.096	1.84
λ_25s_	14.08	34,457	12.99	0.92
DC + α_1_ + λ_25s_	0.16	234	0.088	0.53
SDNN + α_1 _+λ_25s_	0.027	27	0.010	0.38
SDNN	1.85	1,657	0.62	0.34
SDNN + λ_25s_	0.43	372	0.14	0.33
DC + λ_25s_	2.63	1,849	0.70	0.26
DC + α_1_	0.71	289	0.11	0.15
DC	11.36	4,015	1.51	0.13
SDNN + α_1_	0.12	34	0.013	0.11
α_1_ + λ_25s_	0.88	208	0.078	0.089
α_1_	3.80	351	0.132	0.035

Note

Data are the rates and cases for exclusive combinations where no other high‐risk value exists.

Data for no abnormality and for the combination of all of five abnormalities are presented in Table 6 as numbers of abnormalities of 0 and 5, respectively.

* Ratio of observed rate to expected rate. Abbreviations are explained in the footnote to Table 2.

## 4 DISCUSSION

To examine the redundancy between the major mortality risk predictors derived from HRV and HR dynamics, we analyzed the inter‐relationships between the predictors and the tendency of multiple risks clustering in the same person using the 24‐hr Holter ECG big data from the ALLSTAR database. We observed substantial inter‐relationships among the predictors; particularly, a close positive correlation between SDNN and VLF, moderate positive correlations among VLF, DC, and DFA α_1_, and negative correlations of λ_25s_ with the other predictors. The inter‐relationships were able to be visualized by a relationship mapping of predictors (Figure 1). We also observed that multiple high‐risk values of predictors clustered in the same person at a much higher rate than expected rates calculated assuming their mutual independence. Our observations support a high degree of redundancy among their predictive powers.

This study is the first large‐scale systematic analysis of the inter‐relationships of risk predictors derived from HRV and HR dynamics in 24‐hr Holter ECG. There were earlier studies to analyze the relationship between a part of HRV and HR dynamics indices. In the study first to report the prognostic value of DC for post‐AMI mortality, Bauer et al. (Bauer et al., 2006) examined the independent predictive value of DC and SDNN by the Cox proportional hazards regression model. All of their three post‐AMI cohorts, decreased SDNN ≤ 70 ms was a significant univariate mortality risk, but it no longer had independent predictive power when DC ≤ 2.5 ms was entered in the regression models. Although the independence of prognostic power was not examined directly, Huikuri et al. (Huikuri et al., 2000) reported that α_1_ < 0.75 predicted post‐AMI arrhythmic death independently of established clinical risk factors, while none of the time‐ or frequency‐domain HRV indices had independent predictive power. Finally, in a previous study in patients after myocardial infarction (Hayano et al., 2011), we demonstrated that λ_25s_ > 0.6 was a significant predictor of mortality even in the Cox hazards regression models with coexisting SDNN, DC, VLF, and DFA α_1._ These studies suggest both dependency and independency of the predictors, but the entire relationships were unclear from them.

In this study, we used the relationship mapping to visualize the inter‐relationship of all predictors. It was generated so that the distances represent the similarity between predictors. A thorough computer search produced some placements with similar optimality, but they were derivatives of rotation on a plane. Despite its simplicity, predictors that reflect the quality (DFA α_1_ and λ_25s_) and quantity (SDNN and VLF) of HR fluctuations are separated in the upper left and lower right, and those that reflect sympathetic (λ_25s_) (Kiyono et al., 2008) and parasympathetic (SDNN and DFA α_1_) (Huikuri, Perkiomaki, Maestri, & Pinna, 2009) functions are separated vertically. It also suggests the relative independency of λ_25s_ from the other predictors and the similarity between SDNN and VLF. For λ_25s_, higher values represent increased risk (Hayano et al., 2011; Kiyono et al., 2008). Thus, it shows negative correlations with other risk predictors, which are indicated with dotted lines in the relationship mapping.

This study showed that high‐risk values of HRV and HR dynamics tend to cluster in the same person. The probability of multiple high‐risk predictor values appearing in the same person was higher than that expected assuming independence between predictors. Furthermore, the ratio of observed rate to the expected rate increased as the number of risks clustering increased. These indicate that there is an association between predictors and that multiple factors may reflect common features. This is also consistent with the fact that the observed rate of subjects with a single isolated high‐risk value of SDNN, DC, α_1_, or λ_25s_ was lower than the expected rate, indicating that these high‐risk values are unlikely to occur alone. All of these support that there is a high degree of redundancy between the predictors derived from HRV and HR dynamics.

This study has limitations. First, the results of this study were obtained from a single database. Thus, the relationships and mutual dependencies that were found between the predictors derived from HRV and HR dynamics indices may be specific to the ALLSTAR database. They may vary depending on the disease composition of the groups. The ALLSTAR database, however, consists of continuous Holter ECG data collected from all over Japan at the ECG analysis centers. The data collected in this database each year represents approximately 5% of the Holter ECG data recorded annually in Japan. Thus, the subjects in this database are likely to represent the population of patients undergoing Holter ECG in Japan. Second, although we found the close mutual dependencies between the mortality risk predictors in big data, it is unclear whether there is interdependency in the predictive power for actual mortality risk. To confirm the clinical significance of present findings, future studies need to examine the prognostic association of the number of high‐risk values and the individual combinations of the high‐risk values with mortality risk in prospective or retrospective study cohorts.

## 5 CONCLUSIONS

In the ALLSTAR 24‐hr Holter ECG big data, we found that the risk predictors derived from HRV and HR dynamics show a strong tendency for their high‐risk values to cluster in the same person. Our observations support that there is a high degree of redundancy between the predictors.

## 6 ETHICS

This study was a part of the Allostatic State Mapping by Ambulatory ECG Repository (ALLSTAR) project that has been approved by the Ethics Review Committee of Nagoya City University Graduate School of Medical Sciences (No. 709).

## CONFLICTS OF INTEREST

The authors declare no conflict of interest.

## AUTHOR CONTRIBUTIONS

All authors have read and agreed to the published version of the manuscript. Contributed to conceptualization: E.Y. and J.H.; Contributed to methodology: J.H.; Contributed to software: J.H.; Contributed to validation: E.Y., N.U., and M.K.; Contributed to formal analysis: E.Y.; Contributed to investigation: M.K.; Contributed to resources: N.U.; Contributed to data curation: N.U.; Contributed to writing—original draft preparation: J.H.; Contributed to writing—review and editing: E.Y.; Contributed to visualization: J.H.; Contributed to supervision: J.H.; Contributed to project administration: J.H.; Contributed to funding acquisition: J.H.
